# A case report of an improvement in premature ventricular complex–induced cardiomyopathy following continuous positive airway pressure therapy in a patient with severe obstructive sleep apnoea

**DOI:** 10.1093/ehjcr/ytac349

**Published:** 2022-08-22

**Authors:** Togo Sakai, Masao Takemoto, Tokushi Koga, Takuya Tsuchihashi

**Affiliations:** Cardiovascular Center, Steel Memorial Yawata Hospital, 1-1-1 Haruno-machi, Yahatahigashi-ku, Kitakyushu 805-8508, Japan; Cardiovascular Center, Steel Memorial Yawata Hospital, 1-1-1 Haruno-machi, Yahatahigashi-ku, Kitakyushu 805-8508, Japan; Cardiovascular Center, Steel Memorial Yawata Hospital, 1-1-1 Haruno-machi, Yahatahigashi-ku, Kitakyushu 805-8508, Japan; Cardiovascular Center, Steel Memorial Yawata Hospital, 1-1-1 Haruno-machi, Yahatahigashi-ku, Kitakyushu 805-8508, Japan

**Keywords:** Cardiomyopathy, Case report, Continuous positive airway pressure, Obstructive sleep apnoea, Premature ventricular complex

## Abstract

**Background:**

Premature ventricular complexes (PVCs) are the most common arrhythmias observed in patients without structural heart disease (SHD). Frequent PVCs cause left ventricular dilation and dysfunction without SHD, the so-called PVC-induced cardiomyopathy (PIC). Obstructive sleep apnoea (OSA) is a highly prevalent disease worldwide and is strongly associated with arrhythmias including PVCs. PVCs have been reported in up to two-thirds of patients with OSA. Continuous positive airway pressure (CPAP) is a well-established primary treatment modality in patients with moderate-to-severe OSA.

**Case summary:**

We present a 69-year-old male case with severe OSA and an improvement in his PIC following CPAP therapy. He has remained well without any symptoms or arrhythmias for 2 years after the introduction of the CPAP therapy for his OSA.

**Discussion:**

Using CPAP therapy for the treatment of his OSA, we could improve his PIC in accordance with a reduction in frequent PVCs without ablation of the PVCs. Only ablation without CPAP therapy may not be able to completely treat PIC associated with OSA, as in the present case. Thus, physicians should be aware of the possibility of PVCs associated with OSA when examining patients with PVCs. To the best of our knowledge, this is the first report of a case of improvement in PIC following CPAP therapy in a patient with severe OSA. Future investigations should focus on whether CPAP therapy can improve PIC associated with OSA and prevent a progression to heart failure and also result in an improvement in the prognosis.

**Learning points**

- Premature ventricular complexes (PVCs) are the most common arrhythmias observed in patients without structural heart disease and cause left ventricular (LV) dilation and dysfunction, the so-called PVC-induced cardiomyopathy (PIC), which increases the risk of mortality and cardiovascular events.
- Obstructive sleep apnoea (OSA) is a highly prevalent disease worldwide and also causes LV dysfunction and is strongly associated with PVCs, which have been reported in up to two-thirds of patients with OSA.
- In the present case, by using continuous positive airway pressure therapy for his OSA, his PIC improved with a reduction in the PVCs without ablation of his PVCs. Therefore, physicians should be aware of the possibility of PVCs associated with OSA when they examine patients with PVCs.

## Introduction

Premature ventricular complexes (PVCs) are the most common arrhythmias observed in patients without structural heart disease (SHD).^1,2^ Patients with frequent PVCs without SHD are associated with an increased risk of mortality and cardiovascular events.^1^ We previously reported that frequent PVCs caused left ventricular (LV) dilation and dysfunction without SHD,^2^ the so-called PVC-induced cardiomyopathy (PIC).^3^ Elimination of these PVCs by radiofrequency catheter ablation (RFCA) could improve PIC.^2^ Obstructive sleep apnoea (OSA) is a highly prevalent disease worldwide and is strongly associated with arrhythmias including PVCs.^4^ Premature ventricular complexes have been reported in up to two-thirds of patients with OSA, which is significantly higher than the rates reported in patients without OSA.^4^ Continuous positive airway pressure (CPAP) is a well-established primary treatment modality in patients with moderate-to-severe OSA.^4^ Here, we report a case of improvement in PIC in a patient with severe OSA following CPAP therapy.

## Timeline

**Table:** 

1 month prior to admission	Frequent premature ventricular complexes (PVCs) and an elevated brain natriuretic peptide (BNP) level were documented. He experienced daytime sleepiness
At the time of the outpatient department consultation (Day 0)	His neck circumference–height ratio was 0.25. The 12-lead electrocardiogram exhibited frequent PVCs. The echocardiography yielded a mildly reduced left ventricular (LV) ejection fraction (LVEF), LV hypertrophy, and an enlarged LV. His chest X-ray revealed cardiomegaly
Day 7	24 h Holter monitoring showed frequent PVCs
Day 10	Polysomnography revealed severe obstructive sleep apnoea (OSA)
Day 14	No evidence of structural heart disease was revealed by coronary angiography, right heart catheterization, cardiac magnetic resonance imaging, or histology from a right ventricular biopsy Then, a diagnosis of a PVC-induced cardiomyopathy was made. Thus, we planned to perform radiofrequency catheter ablation (RFCA) of his frequent PVCs At the same time, the patient was introduced to continuous positive airway pressure therapy for OSA
1 month after the introduction of the CPAP	The frequent PVCs disappeared after the CPAP therapy. Thus, no RFCA of the PVCs was performed
3 months after the introduction of the CPAP	24 h Holter monitoring showed less PVCs. Furthermore, his BNP level, cardiomegaly, and reduced LVEF improved
2 years after the introduction of the CPAP	He has remained well without any symptoms or arrhythmias

## Case presentation

**Figure 1:**
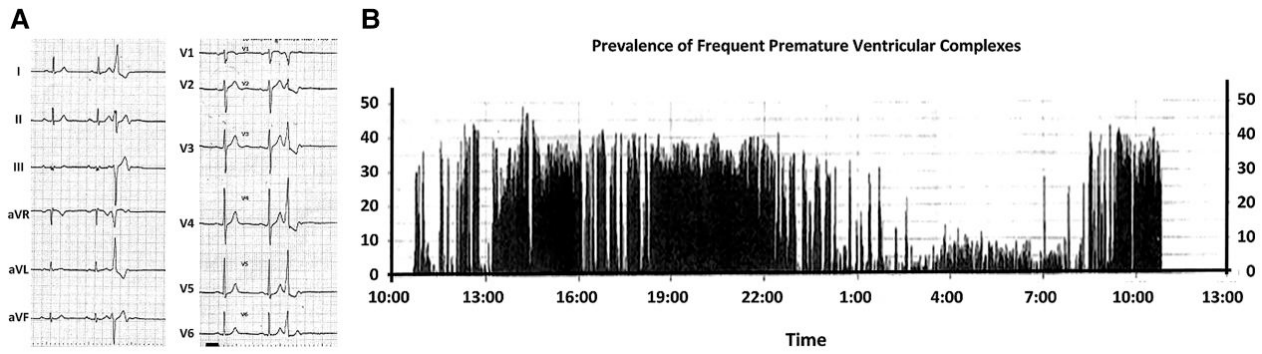
The 12-lead electrocardiograms at the time of the outpatient department consultation (*A*) and histogram of the prevalence of premature ventricular complexes on 24 h Holter monitoring before the introduction of continuous positive airway pressure therapy for his sleep apnoea (*B*).

**Figure 2:**
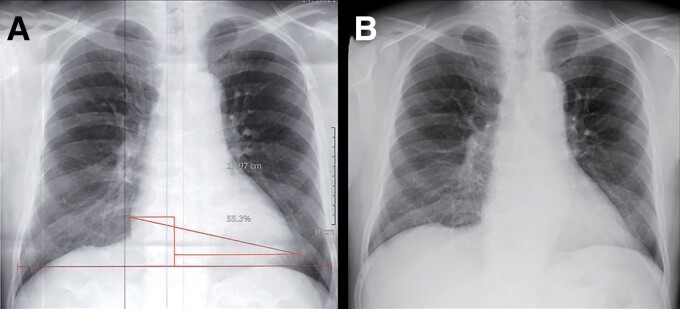
The chest X-ray before (*A*) and 3 months after (*B*) the introduction of continuous positive airway pressure therapy.

**Figure 3:**
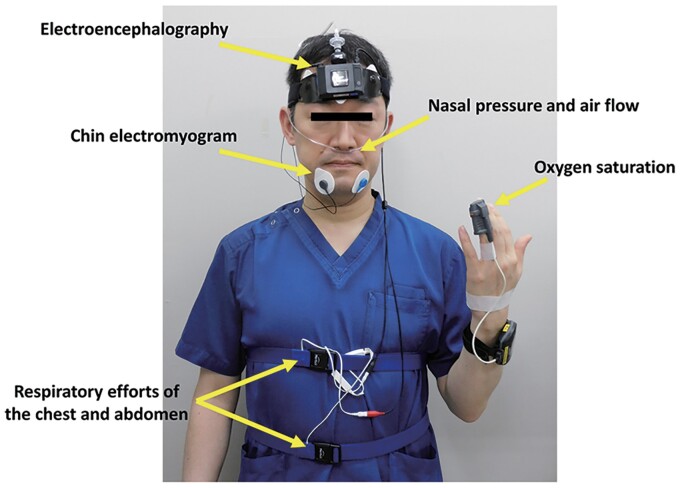
The full polysomnography testing that evaluated the electroencephalography, chin electromyogram, nasal pressure and airflow, respiratory efforts of the chest and abdomen, and oxygen saturation.

**Figure 4:**
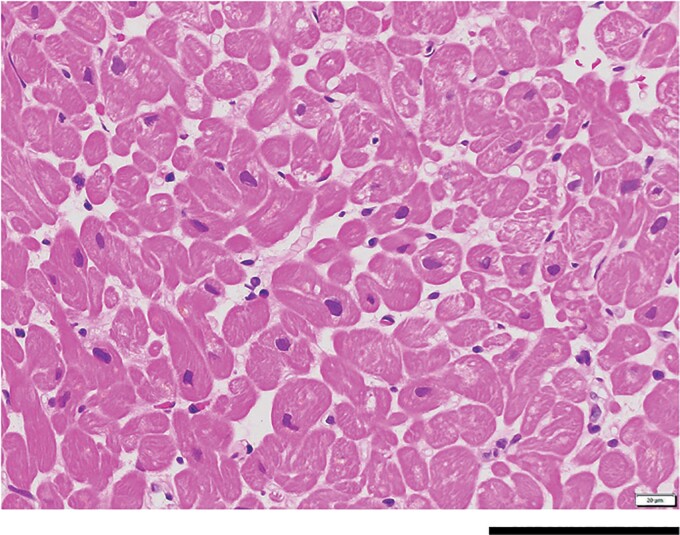
The histological analysis from a right ventricular biopsy revealed cardiac myocyte hypertrophy (haematoxylin and eosin stain, 40×). The black bar indicates 100 µm.

A 69-year-old male was referred to our hospital because of frequent PVCs and an elevated brain natriuretic peptide (BNP) level of 91.2 pg/mL. He experienced daytime sleepiness. He had a history of diabetes mellitus and hypertension and was taking medications including olmesartan 10 mg, azelnidipine 8 mg, vildagliptin 50 mg, and metformin 500 mg daily. At the time of the outpatient department consultation, his blood pressure was 124/64 mmHg and pulse rate 70 beats/min and irregular. Auscultation revealed normal cardiac sounds and normal breath sounds. The lower extremities had no oedema. He was obese, and his body mass index was 28.3 kg/m^2^. His HbA1c was 6.0%. The 12-lead electrocardiogram exhibited normal sinus rhythm, a normal axis, and frequent PVCs (*Figure 1A*). The morphology of the PVCs exhibited a northeast axis, left bundle brunch block (LBBB) in V1, and positive concordant R waves in the precordial leads including V2–V6. Those findings indicated that the probable site of the origin of those PVCs was in the direction of 4:00–6:00 in the left anterior oblique view of the tricuspid valve. The echocardiography yielded a mildly reduced LV ejection fraction (LVEF) of 55% calculated by the Teichholz method, LV hypertrophy of 12 mm, enlarged LV end-diastolic and -systolic dimensions (LVDd/s) of 59 and 43 mm, and normal valvular function (*Table 1*; see Supplementary material online, *Video S1A–C*). His chest X-ray revealed an enlarged cardiothoracic ratio (CTR) of 55% without congestion (*Figure 2A*). The 24 h Holter monitoring showed frequent mono-focal PVCs of 26 589 beats/day. The prevalence of frequent PVCs was observed mainly during the daytime (*Figure 1B*). The %PVCs, which was calculated as (the number of PVCs/number of total heart beats per 24 h) ×  100, was 24.8%. After admission, a full polysomnography test, which evaluated the electroencephalography, chin electromyogram, nasal pressure and airflow, respiratory efforts of the chest and abdomen, and oxygen saturation (*Figure 3*), revealed severe OSA with an apnoea/hypopnoea index (AHI) of 59.5/h, and the lowest arterial oxygen saturation value was 90%. Because his height and neck circumference were 173 and 43 cm, respectively, his neck circumference–height ratio was 0.25.^5^ His coronary angiography (CAG) and right heart catheterization (RHC) were normal. Moreover, the findings from cardiac magnetic resonance imaging (cMRI) and histology from a right ventricular biopsy (RV-Bx) revealed cardiac hypertrophy (*Figure 4*) and no evidence of SHD including cardiac amyloidosis, sarcoidosis, dilated, hypertrophic, and arrhythmogenic right ventricular cardiomyopathy or Fabry disease. Then, a diagnosis of PIC^3^ was made. Thus, we planned to perform RFCA of his frequent PVCs 2 months later because of his work schedule. At the same time, we introduced him to CPAP therapy for his OSA. The prescription of his CPAP was the positive airway pressure, where the airflow was introduced from his nose into the airways to maintain a continuous pressure of 4 cm H_2_O. At the 1-month follow-up after the introduction of the CPAP therapy, surprisingly, his frequent PVCs completely disappeared as confirmed by a 3 min 12-lead electrocardiogram recording. Thus, the RFCA of the PVCs was cancelled. At the 3-month follow-up, his AHI and %PVC on the 24 h Holter monitoring while using CPAP were 2.4/h and 3.0%, respectively. Furthermore, his CTR (*Figure 2B*), LVDd/s, and LVEF improved to 50%, 50 and 29 mm, and 73%, respectively (*Table 1*; see Supplementary material online, *Video S1D–F*). Further, his BNP level normalized was to 8.0 pg/mL. He has remained well without any symptoms or arrhythmias for 2 years after the introduction of the CPAP therapy for the OSA.

**Table 1:** Parameters before and 3 months after continuous positive airway pressure therapy

	Before CPAP therapy	3 months after CPAP therapy
Brain natriuretic peptide (pg/mL)	91.2	8.0
Cardiothoracic ratio of chest X-ray (%)	55	50
Echocardiography		
Left ventricular end-diastolic dimension (mm)	59	50
Left ventricular end-systolic dimension (mm)	43	29
Left ventricular ejection fraction (%)	55	73
Thickness of the interventricular septum (mm)	12	12
Thickness of the posterior wall (mm)	12	12
24 h Holter monitoring		
Total heart beats (beats per 24 h)	107 172	110 330
Premature ventricular complex (beats per day)	26 589	2993
% Premature ventricular complex (%)	24.8	3.0
Premature atrial complex (beats per day)	335	20
Polysomnography		
Apnoea/hypopnoea index (per hour)	59.5	2.4
Lowest SaO_2_ (%)	90	—

CPAP, continuous positive airway pressure; SaO_2_, arterial oxygen saturation.

## Discussion

A surge of data has reproducibly identified a strong association between OSA and arrhythmias including PVCs.^4,6^ Although the mechanism of the OSA causing the PVCs has not been completely elucidated, several potential mechanisms could be raised. Obstructive sleep apnoea is characterized by repetitive upper airway collapses during sleep, resulting in intermittent and repetitive hypoxia and hypercapnia, sleep deprivation, and repetitive intrathoracic pressure changes due to an increased respiratory effort against an occluded upper airway.^4^ Three proposed pathophysiologic pathways responsible for enhanced arrhythmogenesis in OSA have been reported.^4,6^ (i) The immediate pathway includes intermittent and repetitive hypoxia and hypercapnia eliciting an enhanced parasympathetic activation during OSA following sympathetic surges subsequent to OSA^6,7^ and an alternation in the ventricular repolarization^4^ and endocardial Ca^2+^ channel expression.^8^ These sequential autonomic alterations lead to enhanced arrhythmia susceptibility,^6^ contributing to an increase in the number of PVCs during sleep. On the other hand, sleep deprivation also increases the sympathetic nerve activity and significantly increases the number of PVCs during the daytime^7^ as in the present case. (ii) The intermediate pathway includes increased systemic inflammation, formation of reactive oxygen species contributing to an altered Ca^2+^ channel activation,^4^ and vascular dysfunction.^4,6^ These actions predispose to cardiovascular disease development and possibly an increased arrhythmia propensity.^6^ However, in this case, the patient had no cardiovascular disease. (iii) The chronic pathway includes cardiac electrical and structural remodelling such as fibrosis and LV hypertrophy, leading to electrophysiologic alterations predisposing to arrhythmogenesis.^6^ In another report, a high AHI was independently associated with an increased prevalence of PVCs not only at night but also during daytime,^9^ as in this case. Those factors caused by OSA may have predisposed to triggered and abnormal automaticity and, finally, contributed to an augmented arrhythmic propensity for PVCs. In view of these findings, in this present case, the sleep deprivation and cardiac electrical and structural remodelling associated with OSA might be important factors to produce PVCs during the daytime.

The diagnosis of PIC is presumptively based on the presence of frequent PVCs, an existing cardiomyopathy, and a lack of findings of SHD and is verified when the elimination of the PVCs results in the resolution of the cardiomyopathy.^3^ In this present case, his CAG, RHC, cMRI, and histology from an RV-Bx revealed no evidence of SHD. Further, the elimination of the PVCs down to 3.0% steadily resulted in the resolution of the LV dilation and dysfunction.^10^ Thus, a diagnosis of PIC was made. The frequent PVCs in this patient demonstrated an LBBB pattern, which can cause dyssynchrony and disrupt the squeezing effects and torsional deformation of the LV.^2^ Those effects may finally cause the PIC.^2^ Obstructive sleep apnoea also has been reported to cause a decrease in the LV function and the development of LV hypertrophy.^6^ Because the neck circumference–height ratio in this patient was 0.25, he was at high risk for OSA.^5^ The repetitive intrathoracic pressure changes, which caused the development of sympathetic nerve activity and cardiac electrical remodeling associated with OSA, may cause the development not only of LV dysfunction but also of cardiac structural remodelling such as fibrosis and LV hypertrophy.^6^ Thus, despite their shared common risk factors, frequent PVCs and OSA subtypes may exacerbate each other and accelerate cardiac structural changes including LV dysfunction and hypertrophy.

Continuous positive airway pressure is a treatment that can efficiently reduce the AHI and deliver positive pressure through a mask to maintain the opening of the upper airway during sleep in patients with moderate-to-severe OSA. Although the definitive evidence and mechanisms of the efficacy of CPAP for PVCs have still not been established, it may be important for suppressing and improving the three proposed pathophysiologic pathways, described above.

The BNP level is known to be a sensitive indicator and predictor of heart failure (HF) and PVCs.^11^ In this present case, CPAP therapy could steadily normalize the BNP level from 91.2 to 8.0 pg/mL, indicating the prevention of the progression of the HF associated with PIC resulting from OSA.

## Conclusions

Fortunately, using CPAP therapy, we could improve PIC in accordance with a reduction in the frequent PVCs and AHI resulting from the treatment of OSA, and it provided a good clinical course without RFCA of the PVCs. Only RFCA without CPAP therapy may not be able to completely treat PIC associated with OSA, as in the present case. Thus, physicians should be aware of the possibility of PVCs associated with OSA when examining patients with PVCs. To the best of our knowledge, this is the first report of a case of improvement in PIC following CPAP therapy in a patient with severe OSA. Future investigations should focus on whether CPAP therapy could improve PIC associated with OSA and prevent the progression to HF, including the potential for an improvement in the prognosis.

## Supplementary Material

ytac349_Supplementary_Data

## Lead author biography

Dr Togo Sakai received his MD degree from Saga University (Saga, Japan) in 2013. Since 2020, he has been working as a cardiologist at the Steel Memorial Yawata Hospital. His area of medical interest is heart failure, arrhythmias, sleep disorder, and coronary artery disease.
