# Supplementary material for: A case report of an improvement in premature ventricular complex–induced cardiomyopathy following continuous positive airway pressure therapy in a patient with severe obstructive sleep apnoea
Source: Eur Heart J Case Rep. 2022 Aug 22;6(9):ytac349. doi: 10.1093/ehjcr/ytac349 (PMC9446675; doi:10.1093/ehjcr/ytac349)
Supplement: ytac349_Supplementary_Data [file ytac349_supplementary_data.zip › renamed_a36e0.pptx]

## Slide 1
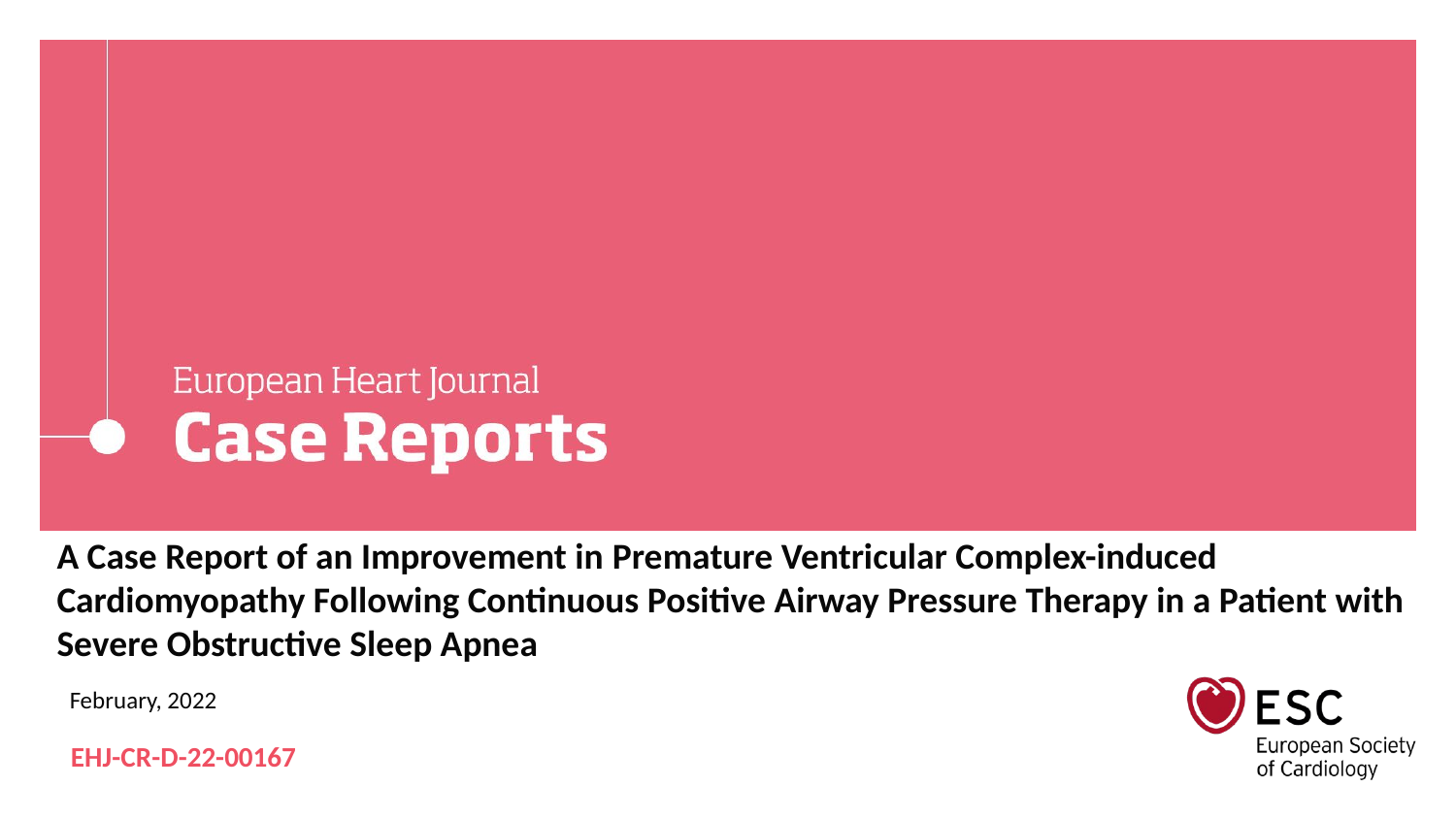

# A Case Report of an Improvement in Premature Ventricular Complex-induced Cardiomyopathy Following Continuous Positive Airway Pressure Therapy in a Patient with Severe Obstructive Sleep Apnea
February, 2022
EHJ-CR-D-22-00167

## Slide 2
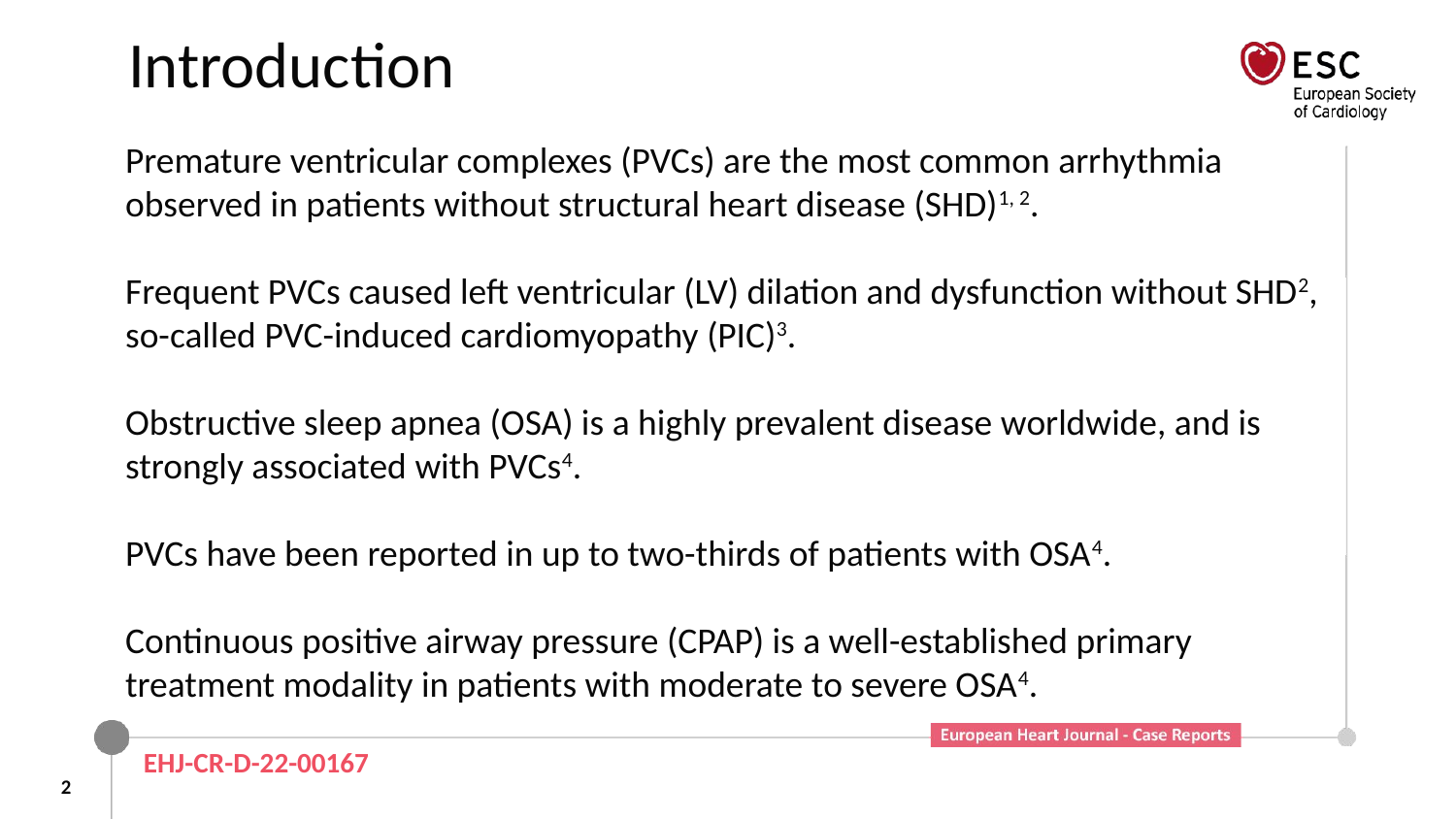

# Introduction
Premature ventricular complexes (PVCs) are the most common arrhythmia observed in patients without structural heart disease (SHD)1, 2.
Frequent PVCs caused left ventricular (LV) dilation and dysfunction without SHD2, so-called PVC-induced cardiomyopathy (PIC)3.
Obstructive sleep apnea (OSA) is a highly prevalent disease worldwide, and is strongly associated with PVCs4.
PVCs have been reported in up to two-thirds of patients with OSA4.
Continuous positive airway pressure (CPAP) is a well-established primary treatment modality in patients with moderate to severe OSA4.
EHJ-CR-D-22-00167
2

## Slide 3
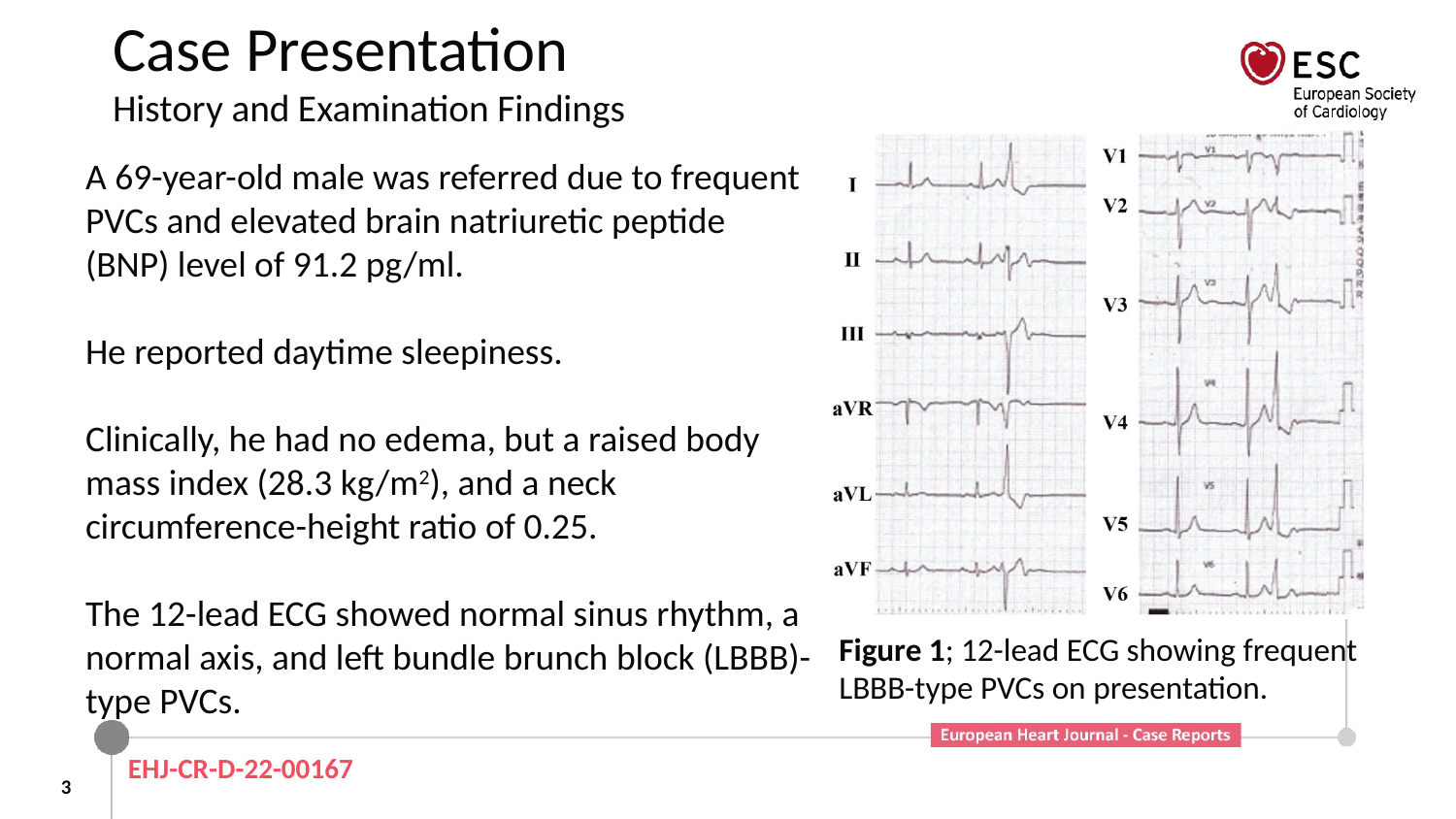

# Case Presentation History and Examination Findings
A 69-year-old male was referred due to frequent PVCs and elevated brain natriuretic peptide (BNP) level of 91.2 pg/ml.
He reported daytime sleepiness.
Clinically, he had no edema, but a raised body mass index (28.3 kg/m2), and a neck circumference-height ratio of 0.25.
The 12-lead ECG showed normal sinus rhythm, a normal axis, and left bundle brunch block (LBBB)-type PVCs.
Figure 1; 12-lead ECG showing frequent LBBB-type PVCs on presentation.
EHJ-CR-D-22-00167
3

## Slide 4
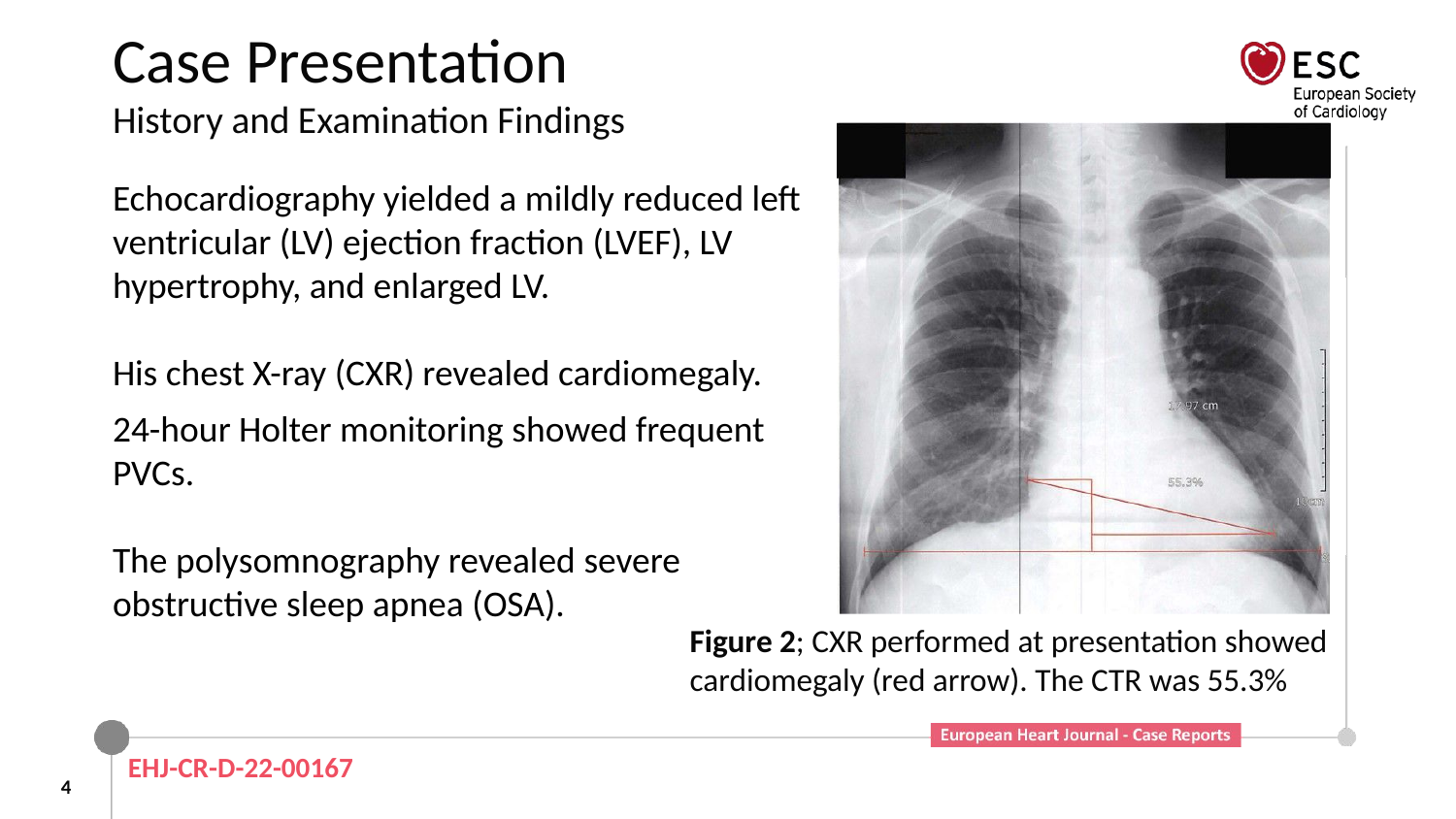

# Case Presentation History and Examination Findings
Echocardiography yielded a mildly reduced left ventricular (LV) ejection fraction (LVEF), LV hypertrophy, and enlarged LV.
His chest X-ray (CXR) revealed cardiomegaly.
24-hour Holter monitoring showed frequent PVCs.
The polysomnography revealed severe obstructive sleep apnea (OSA).
Figure 2; CXR performed at presentation showed cardiomegaly (red arrow). The CTR was 55.3%
EHJ-CR-D-22-00167
4

## Slide 5
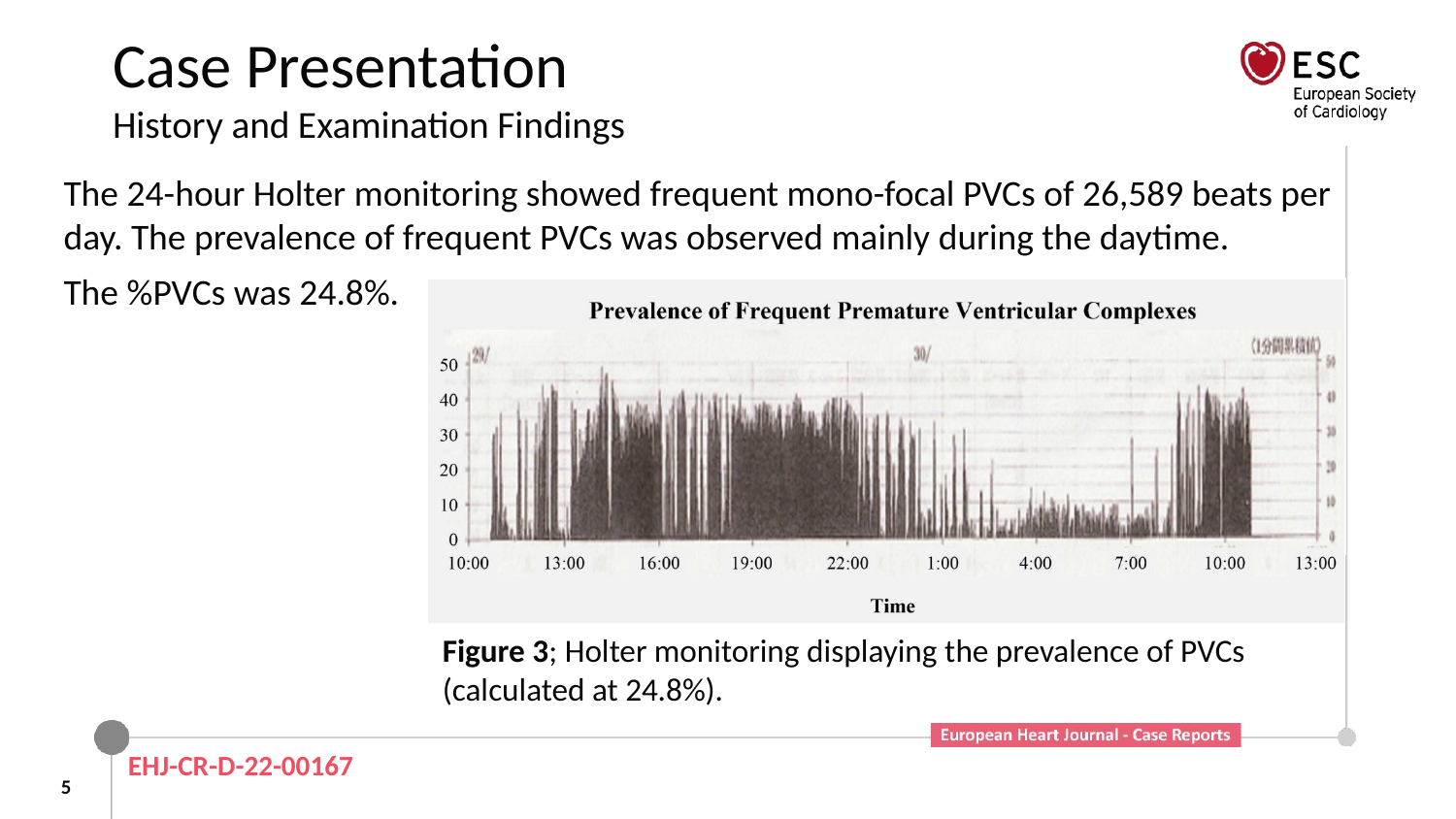

# Case Presentation History and Examination Findings
The 24-hour Holter monitoring showed frequent mono-focal PVCs of 26,589 beats per day. The prevalence of frequent PVCs was observed mainly during the daytime.
The %PVCs was 24.8%.
Figure 3; Holter monitoring displaying the prevalence of PVCs (calculated at 24.8%).
EHJ-CR-D-22-00167
5

## Slide 6
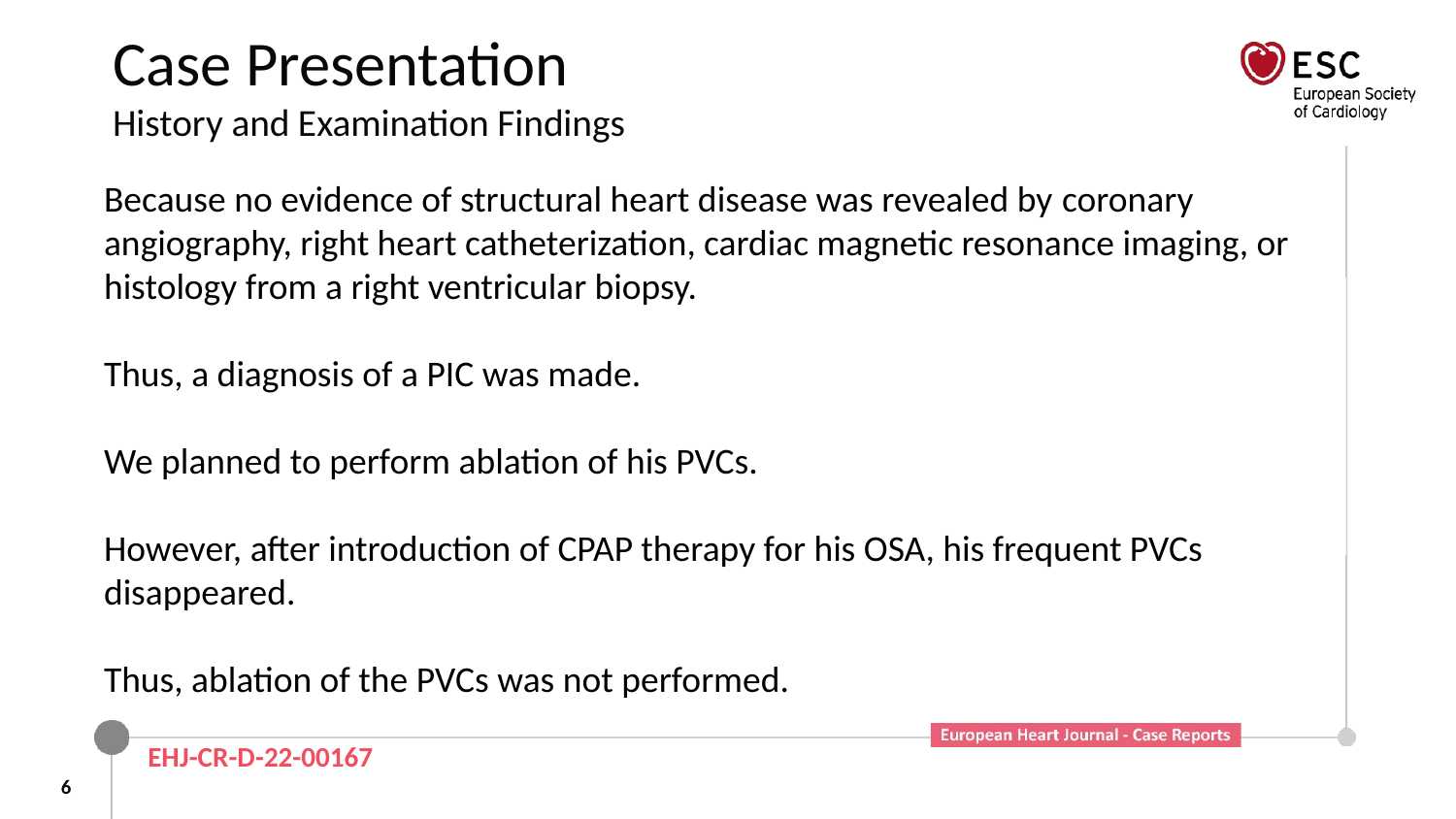

# Case Presentation History and Examination Findings
Because no evidence of structural heart disease was revealed by coronary angiography, right heart catheterization, cardiac magnetic resonance imaging, or histology from a right ventricular biopsy.
Thus, a diagnosis of a PIC was made.
We planned to perform ablation of his PVCs.
However, after introduction of CPAP therapy for his OSA, his frequent PVCs disappeared.
Thus, ablation of the PVCs was not performed.
EHJ-CR-D-22-00167
6

## Slide 7
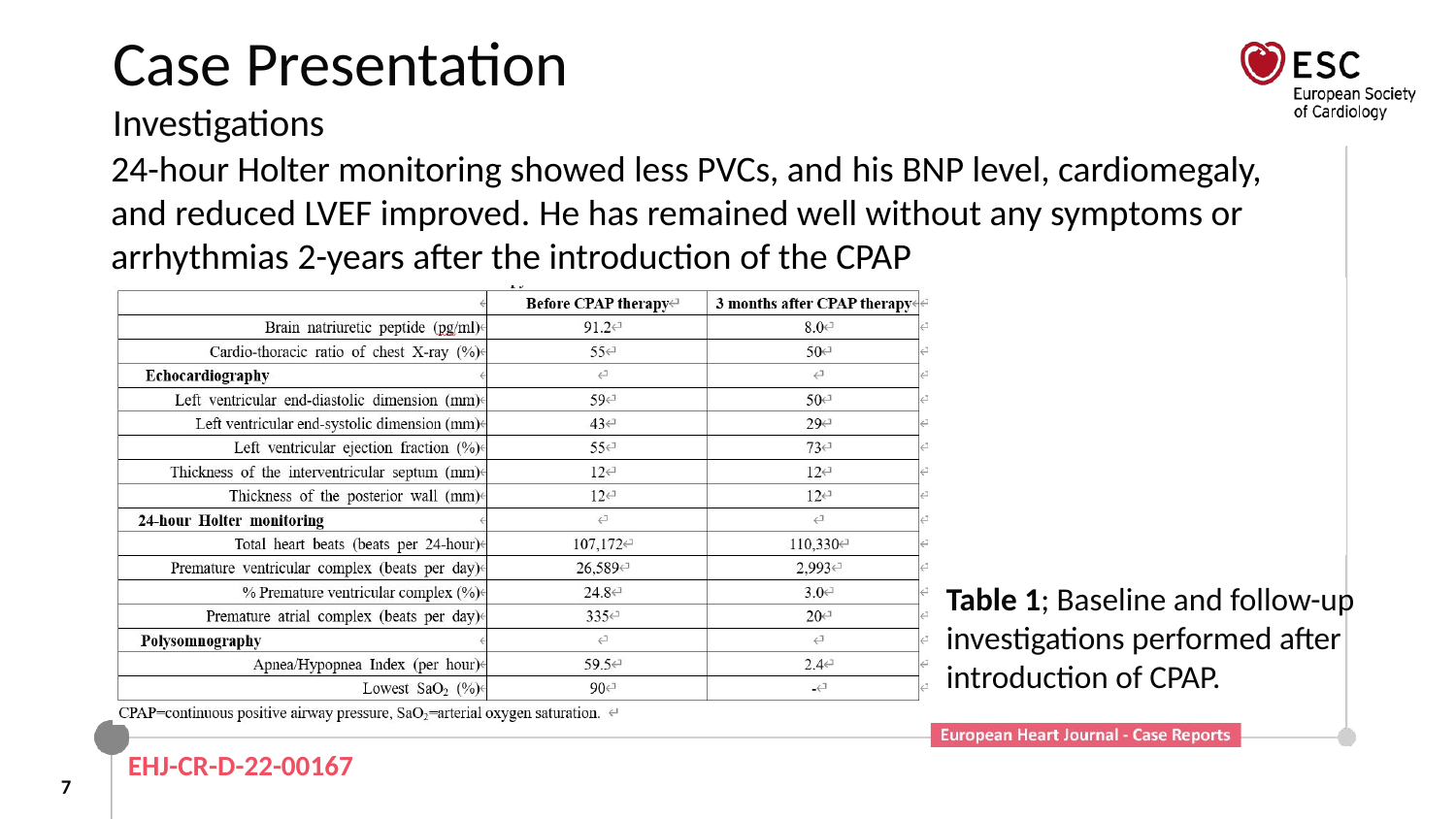

# Case Presentation Investigations
24-hour Holter monitoring showed less PVCs, and his BNP level, cardiomegaly, and reduced LVEF improved. He has remained well without any symptoms or arrhythmias 2-years after the introduction of the CPAP
Table 1; Baseline and follow-up investigations performed after introduction of CPAP.
EHJ-CR-D-22-00167
7

## Slide 8
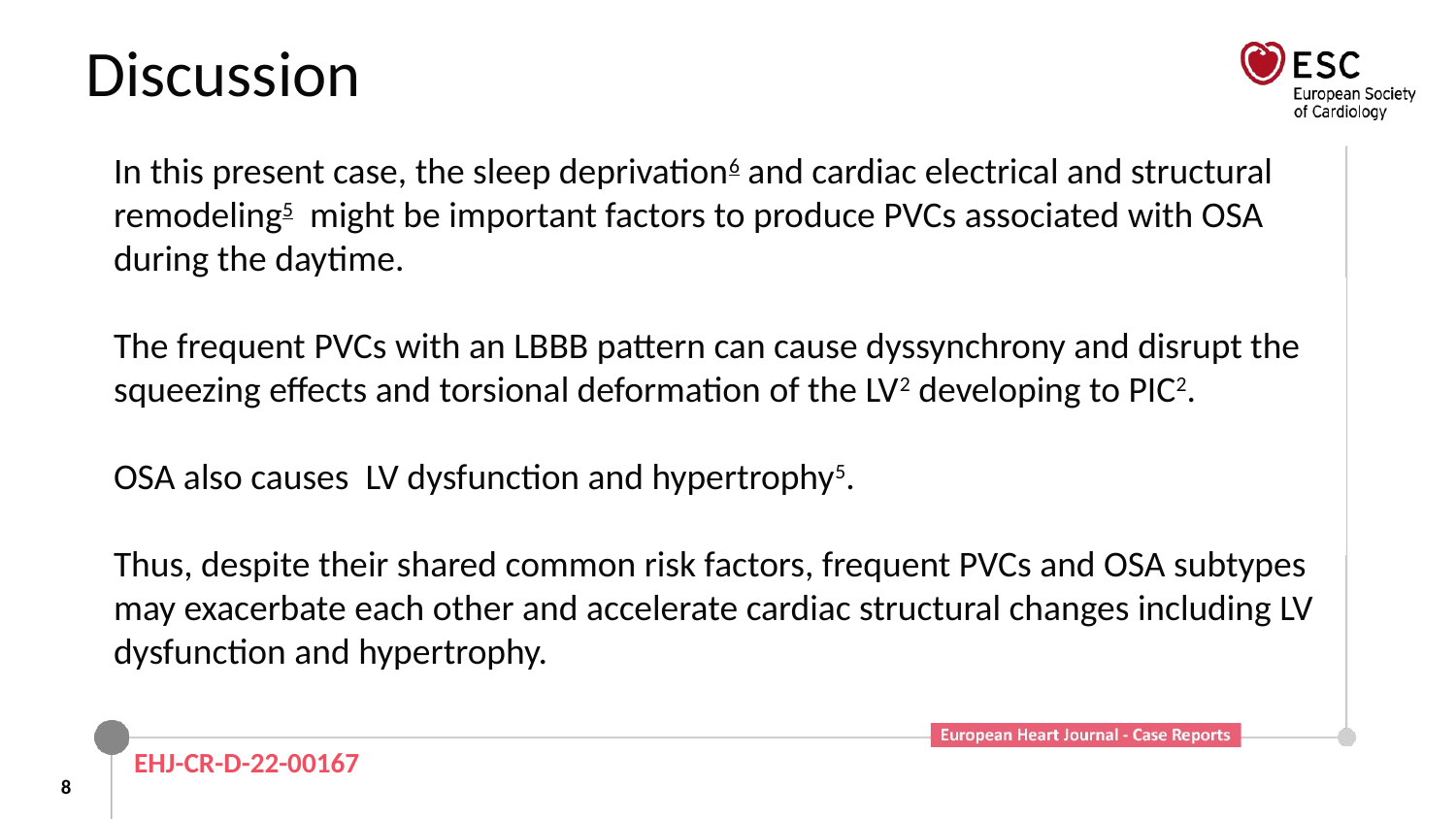

# Discussion
In this present case, the sleep deprivation6 and cardiac electrical and structural remodeling5 might be important factors to produce PVCs associated with OSA during the daytime.
The frequent PVCs with an LBBB pattern can cause dyssynchrony and disrupt the squeezing effects and torsional deformation of the LV2 developing to PIC2.
OSA also causes LV dysfunction and hypertrophy5.
Thus, despite their shared common risk factors, frequent PVCs and OSA subtypes may exacerbate each other and accelerate cardiac structural changes including LV dysfunction and hypertrophy.
EHJ-CR-D-22-00167
8

## Slide 9
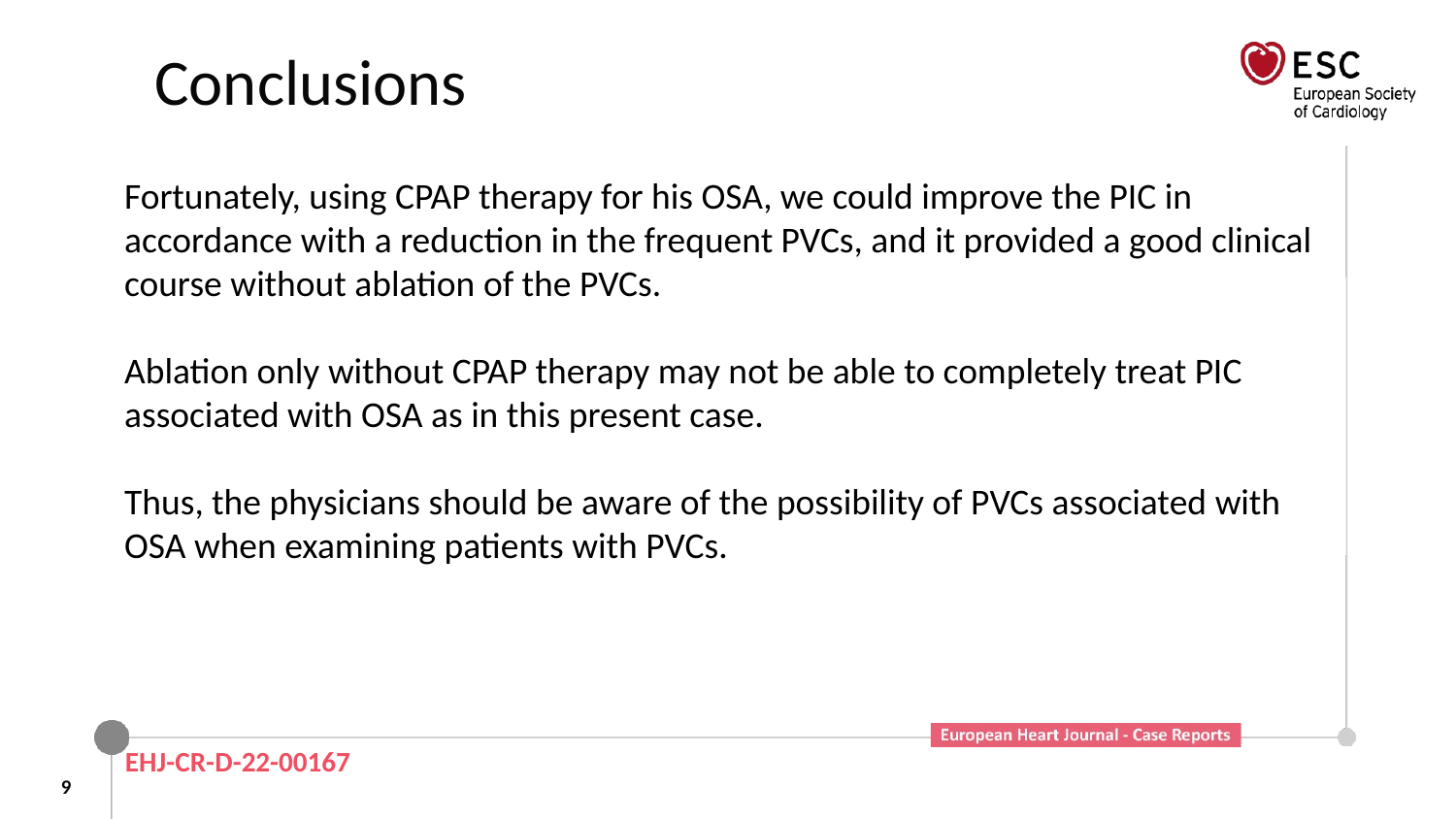

# Conclusions
Fortunately, using CPAP therapy for his OSA, we could improve the PIC in accordance with a reduction in the frequent PVCs, and it provided a good clinical course without ablation of the PVCs.
Ablation only without CPAP therapy may not be able to completely treat PIC associated with OSA as in this present case.
Thus, the physicians should be aware of the possibility of PVCs associated with OSA when examining patients with PVCs.
EHJ-CR-D-22-00167
9

## Slide 10
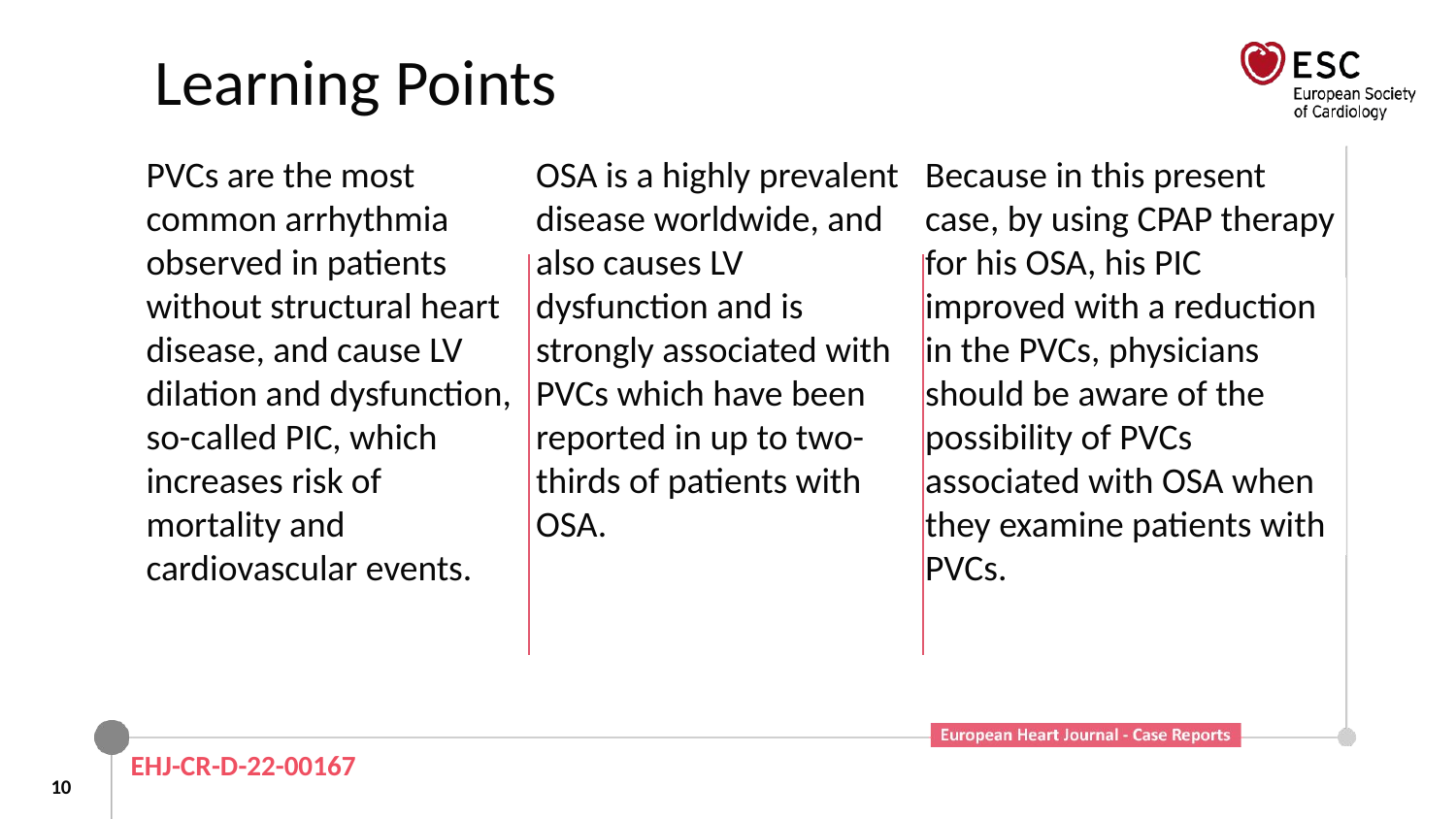

# Learning Points
PVCs are the most common arrhythmia observed in patients without structural heart disease, and cause LV dilation and dysfunction, so-called PIC, which increases risk of mortality and cardiovascular events.
OSA is a highly prevalent disease worldwide, and also causes LV dysfunction and is strongly associated with PVCs which have been reported in up to two-thirds of patients with OSA.
Because in this present case, by using CPAP therapy for his OSA, his PIC improved with a reduction in the PVCs, physicians should be aware of the possibility of PVCs associated with OSA when they examine patients with PVCs.
EHJ-CR-D-22-00167
10

## Slide 11
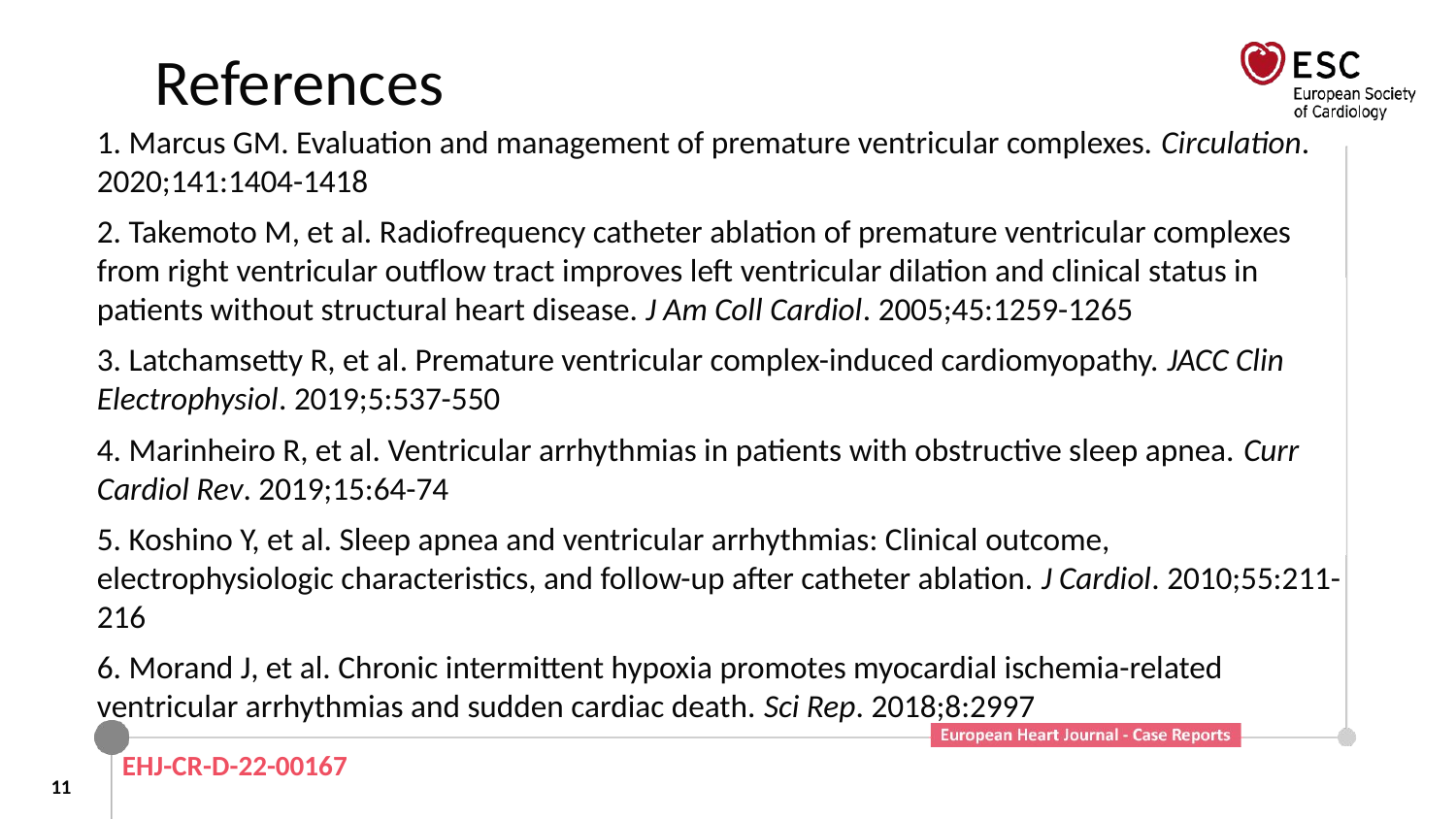

# References
1. Marcus GM. Evaluation and management of premature ventricular complexes. Circulation. 2020;141:1404-1418
2. Takemoto M, et al. Radiofrequency catheter ablation of premature ventricular complexes from right ventricular outflow tract improves left ventricular dilation and clinical status in patients without structural heart disease. J Am Coll Cardiol. 2005;45:1259-1265
3. Latchamsetty R, et al. Premature ventricular complex-induced cardiomyopathy. JACC Clin Electrophysiol. 2019;5:537-550
4. Marinheiro R, et al. Ventricular arrhythmias in patients with obstructive sleep apnea. Curr Cardiol Rev. 2019;15:64-74
5. Koshino Y, et al. Sleep apnea and ventricular arrhythmias: Clinical outcome, electrophysiologic characteristics, and follow-up after catheter ablation. J Cardiol. 2010;55:211-216
6. Morand J, et al. Chronic intermittent hypoxia promotes myocardial ischemia-related ventricular arrhythmias and sudden cardiac death. Sci Rep. 2018;8:2997
EHJ-CR-D-22-00167
11
